# Metabolic reprogramming based on RNA sequencing of gemcitabine-resistant cells reveals the FASN gene as a therapeutic for bladder cancer

**DOI:** 10.1186/s12967-024-04867-8

**Published:** 2024-01-13

**Authors:** Lijie Zhou, Kaixuan Du, Yiheng Dai, Youmiao Zeng, Yongbo Luo, Mengda Ren, Wenbang Pan, Yuanhao Liu, Lailai Zhang, Ronghui Zhu, Dapeng Feng, Fengyan Tian, Chaohui Gu

**Affiliations:** 1 Department of Urology, First Affiliated Hospital of Zhengzhou University, Zhengzhou, Henan China; 2 Department of Urology, Henan Institute of Urology and Zhengzhou Key Laboratory for Molecular Biology of Urological Tumor Research, First Affiliated Hospital of Zhengzhou University, Zhengzhou, Henan China; 3 Unit of Day Surgery Center, First Affiliated Hospital of Zhengzhou University, Zhengzhou, China; 4 Department of Pediatrics, The First Affiliated Hospital of Zhengzhou University, Zhengzhou, China

## Abstract

Bladder cancer (BLCA) is the most frequent malignant tumor of the genitourinary system. Postoperative chemotherapy drug perfusion and chemotherapy are important means for the treatment of BLCA. However, once drug resistance occurs, BLCA develops rapidly after recurrence. BLCA cells rely on unique metabolic rewriting to maintain their growth and proliferation. However, the relationship between the metabolic pattern changes and drug resistance in BLCA is unclear. At present, this problem lacks systematic research. In our research, we identified and analyzed resistance- and metabolism-related differentially expressed genes (RM-DEGs) based on RNA sequencing of a gemcitabine-resistant BLCA cell line and metabolic-related genes (MRGs). Then, we established a drug resistance- and metabolism-related model (RM-RM) through regression analysis to predict the overall survival of BLCA. We also confirmed that RM-RM had a significant correlation with tumor metabolism, gene mutations, tumor microenvironment, and adverse drug reactions. Patients with a high drug resistance- and metabolism-related risk score (RM-RS) showed more active lipid synthesis than those with a low RM-RS. Further in vitro and in vivo studies were implemented using Fatty Acid Synthase (FASN), a representative gene, which promotes gemcitabine resistance, and its inhibitor (TVB-3166) that can reverse this resistance effect.

**Supplementary Information:**

The online version contains supplementary material available at 10.1186/s12967-024-04867-8.

## Statement of Significance

The RM-RM aid to accurately predict survival rates and are used to help guide BLCA patients to choose the appropriate treatment option, and by inhibiting the fatty acid synthesis pathway involved in FASN a potential therapeutic strategy for BLCA is presented

**Supplementary Information:**

The online version contains supplementary material available at 10.1186/s12967-024-04867-8.

## Introduction

BLCA is the most frequent diagnosed malignant tumor of the genitourinary system [1]. Although unprecedented progress has been made in early diagnosis of BLCA tumors and multiple treatment options have been established (such as surgery and intravesical BCG) for primary BLCA in the past decade, the high recurrence rate and poor prognosis of this disease still remains invasive, especially for individuals diagnosed with muscle-invasive BLCA [2]. Long-term regular infusion of chemotherapy drugs after surgery is the most effective preventive care to reduce the recurrence of nonmuscle-invasive BLCA, while postoperative chemotherapy is the first-line therapeutic strategy for BLCA with progression. However, due to intratumor heterogeneity and chemotherapy resistance, the efficacy of the current treatment methods for BLCA is largely limited, and the 5 year of survival rate is still unsatisfactory [3, 4]. The median survival rate of patients who received the most common chemotherapy regimen, gemcitabine and cisplatin (GC scheme), was limited to a timespan of 14 months [5]. Therefore, exploring the mechanism of tumor resistance is crucial for discovering new targets for chemotherapy sensitivity and promoting the progress of precision therapy.

It is well known that metabolic reprogramming is a hallmark of cancer [6]. More and more evidence has shown that cancer cell response to treatment is controlled by the metabolic state, suggesting that metabolism-related pathways could overcome resistance through the controlled metabolic state [7]. Li Y et al. reported that the GLUT1/ALDOB/G6PD axis regulate glucose metabolism reprogramming and promotes chemotherapy resistance in pancreatic cancer [8]. Zhou et al. proved the important role of lipid metabolism during the process of cancer resistance in the treatment of castration resistant prostate cancer [9]. Wong TL et al. confirmed that SCD1 promotes the formation of lipid droplets to target 5-fluorouracil and cisplatin resistance in gastric cancer [10]. Solanki S et al. identified amino acid metabolism to be essential in the cellular reprogramming process of chemoresistance in chemotherapeutic-resistant patients diagnosed with colon cancer [11]. However, there are few studies on metabolism in chemotherapy-related pathways resistance in BLCA. BLCA cells rely on their own unique metabolic transformation to maintain the energy needed for their growth and proliferation [12]. At the same time, the metabolism of bladder cancer is characterized by increased fatty acid synthesis and the phosphopentose pathway, and decreased AMP-activated protein kinase and Krebs cycle activity. The mRNA modification of PKM2 promotes glucose metabolism in BLCA [13]. Afonso J et al. described that glucose metabolism could be a target to improve BLCA immunotherapy [14]. However, there is a lack of systematic analysis on the relationship between the potential mechanism of chemotherapy resistance and metabolic recombination in BLCA.

In this study, we obtained drug-resistant differentially expressed by RNA sequencing of the established gemcitabine-resistant bladder cancer cell line, combined with MRGs, and then established and justified a prognostic model that is found in several BLCA databases through Cox and LASSO regression analysis. Our studies found that this model is a very accurate predictor of overall survival (OS), and is significantly related to metabolic reprogramming, gene mutation, and the tumor microenvironment. In addition, FASN was considered the representative gene of RM-RM. We proved that FASN promoted BLCA gemcitabine resistance, while TVB-3166, an inhibitor of FASN, reversed BLCA gemcitabine resistance in vitro and in vivo.

In summary, we will provide a new model for predicting the survival and therapeutic strategies for BLCA patients.

## Results

### Identification of gemcitabine resistance and metabolism-related differentially expressed genes in BLCA

**Fig. 1:**
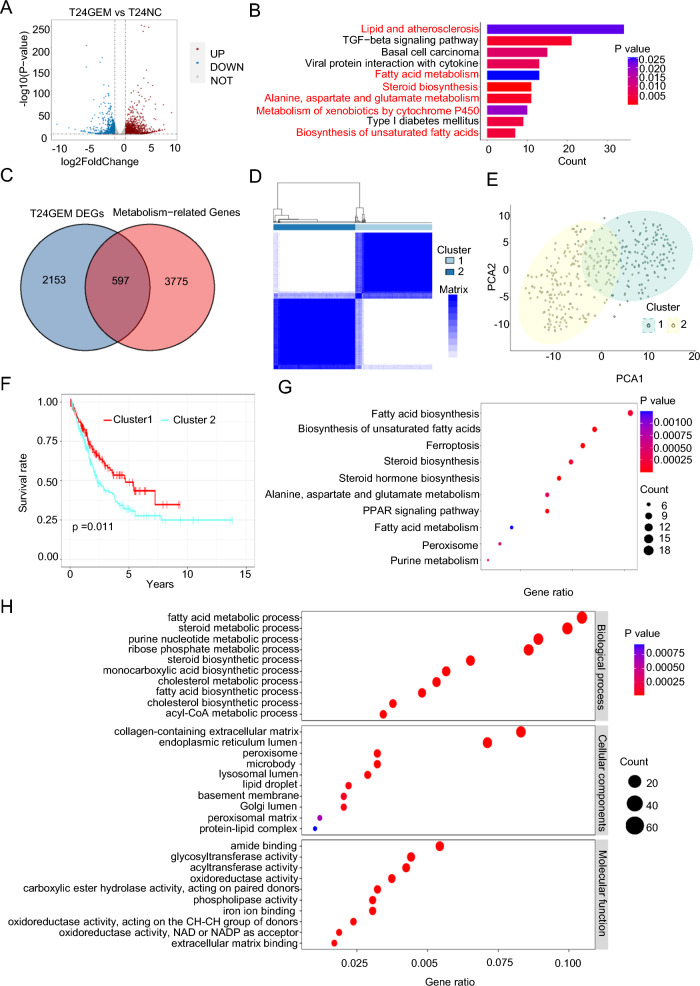
Identification of Gemcitabine Resistance and Metabolism-Related Differentially Expressed Genes in BLCA. **A** By comparing T24 gemcitabine-resistant cells with nonresistant cells, a volcano map of differentially expressed genes (DEGs) was drawn. Blue represents downregulated genes, and red represents upregulated genes in drug-resistant cells. p < 0.05, |FC|> 2 **B** Kyoto Encyclopedia of Genes and Genomes (KEGG) enrichment analysis of DEGs. Adjusted p < 0.01, p < 0.05 **C** Venn diagram for the Resistance and Metabolism-related Differentially Expressed Genes (RM-DEGs) **D**, **E**, **F** Consensus clustering of TCGA BLCA cohorts based on the RM-DEGs. Consensus matrix for optimal k = 2. The optimal k = 2 for the principal component analysis (PCA) database. Kaplan‒Meier analysis was used to analyze the overall survival (OS) curve of patients in different groups. **G**, **H** RM-DEGs were concentrated and analyzed by Gene Ontology (GO) and Kyoto Encyclopedia of Genes and Genomes (KEGG). Adjusted p < 0.01 and p < 0.05

With the purpose of studying the molecular biological changes in BLCA cells after gemcitabine resistance, we obtained drug-resistant differential genes by RNA sequencing of the established gemcitabine-resistant BLCA cell line (Fig. 1A). Subsequently, we performed GO (Additional file 1: Figure S1A) and KEGG (Fig. 1B) enrichment analyses. The KEGG results revealed that these gene were related to lipids, fatty acid metabolism, cholesterol metabolism and amino acid metabolism. The top ten GO terms were enriched in cholesterol synthesis and metabolism, the response to the lipid, extracellular matrix, and the response to the chemical, etc.

With the purpose of studying the metabolic changes of in BLCA cells after gemcitabine resistance, we further screened 597 resistance- and metabolism-related differentially expressed Genes (RM-DEGs, Fig. 1C). Patients with BLCA were divided into two subgroups on the basis of RM-DEG expression in the TCGA BLCA database by consensus clustering (Additional file 1: Figure S1B–E). The two subgroups included 177 and 189 patients (Fig. 1D, E), respectively. Through KM analysis, we discovered noteworthy differences in OS between the two subgroups. (Fig. 1F). Then, we carried out further functional analysis of RM-DEGs. The KEGG analysis revealed that the RM-DEGs were significantly linked with fatty acid biosynthesis, steroid biosynthesis, the PPAR signaling pathway, ferroptosis and other metabolic pathways (Fig. 1G). The top ten GO enrichment pathways mainly included the following aspects (Fig. 1H): biological process included fatty acid, steroid and purine nucleotide metabolism procedure; cellular components included extracellular matrix, endoplasmic reticulum and lipid droplet; molecular function included glycosyltransferase activity, oxidoreductase activity and extracellular matrix binding activity. In short, the above consequences revealed that metabolic reprogramming of tumors play a significant role in drug resistance progression and on the overall survival of BLCA Patients.

### Establishment of the RM-RM to predict the OS of BLCA patients

**Fig. 2:**
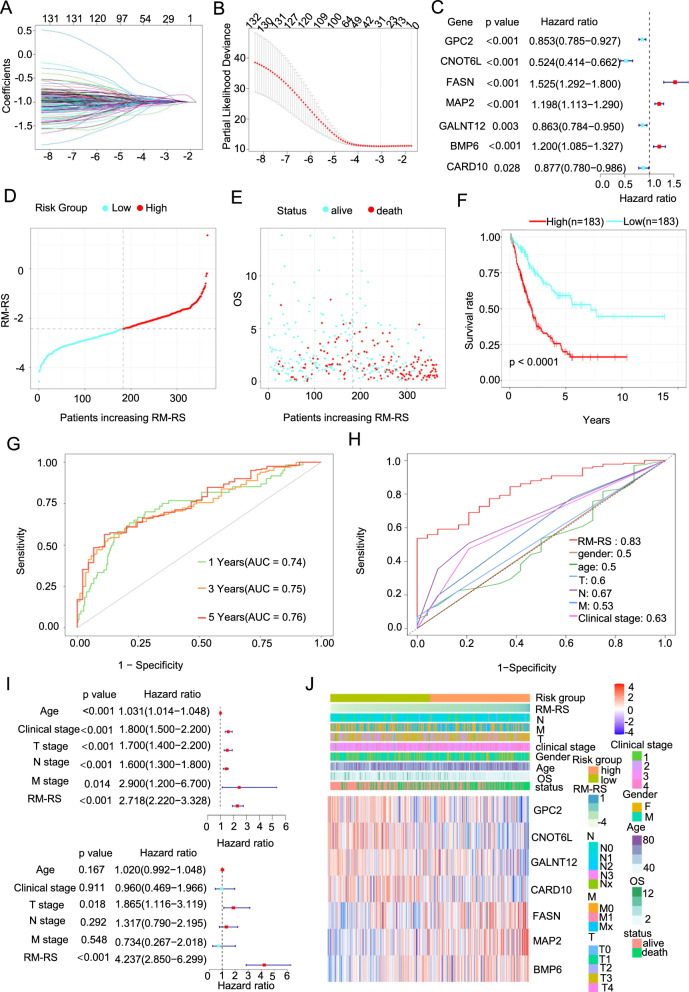
Establishment of an RM-RM to Predict the OS of BLCA Patients. **A**, **B** Least absolute shrinkage and selection operator (LASSO) Cox regression of OS-related key drug resistance and metabolism-related differentially expressed genes (RM-DEGs). **C** Multivariate Cox regression analysis was performed on seven key genes obtained based on cross validation and the minimum partial likelihood deviance. **D** The drug resistance and metabolism-related risk score (RM-RS) distribution of the cancer genome atlas (TCGA) BLCA. The median was the dividing line, blue was the low RM-RS subgroup, and red was the high RM-RS subgroup. **E** The overall survival distribution of the two subgroups. Blue represents alive, while red represent death. **F** Kaplan‒Meier analysis of overall survival (OS) curves of TCGA BLCA patients in the two subgroups. **G** The receiver operating characteristic (ROC) curves of 1-, 3-, and 5 year OS of patients in TCGA BLCA database was predicted based on RM-RS. **H** Comparison of ROC curves between RM-RS and clinical features. **I** Univariate and multivariate Cox regression analyses of RM-RS and clinical features. **J** The heatmap of RM-RM 7 component gene expression in the TCGA BLCA database, including RM-RS and clinical features

First, by analyzing the relationship between a single gene and the OS of BLCA, we selected 134 OS-related RM-DEGs (P < 0.05, Additional file 2: Form S1). As shown in Additional file 1: Figure S2A, most of the OS-related RM-DEGs were closely correlated, indicating that the progression of BLCA resistance is a whole metabolic rearrangement. Then, we performed LASSO Cox regression analysis on OS-related RM-DEGs to further narrow the range of the primary genes that predict prognostic risk. As shown in Fig. 2A, B**, **28 genes were obtained by removing any overfitting data to avoid the minimum likelihood of bias. Finally, through the multivariable Cox regression analysis, we obtained 7 independent prognostic genes. As shown in Fig. 2C**,** the hazard ratio and 95% confidence interval of the four genes were greater than 1, and the remaining three genes were less than 1. This suggested that GPC2, CNOT6L, GALNT12 and CARD10 were independent protective factors and that FASN, MAP2 and BMP6 were independent risk factors. Through the gene index obtained from multivariable Cox regression analysis, we constructed RM-RM and drug Resistance and Metabolism-Related risk Score (RM-RS) = (− 0.16) *GPC2 gene expression + (− 0.65) * CNOT6L gene expression + 0.42 * FASN gene expression + 0.18 * MAP2 gene expression + (− 0.15) * GALNT12 gene expression + 0.18 * BMP6 gene expression + (− 0.13) * CARD10 gene expression. After calculating the risk score, we divided 366 BLCA patients into a high-hazard cluster and a low-hazard cluster in accordance with the median of the RM-RS (Fig. 2D). As shown in Fig. 2E, the OS of the high-hazard cluster was apparently shorter than that of the low-hazard cluster. Compared with patients subjected to low RM-RS, patients with high RM-RS usually have a poor prognosis (Fig. 2F). The results showed that the area under the curves (AUCs) were 0.74,0.75, and 0.76 in the first, third, and fifth years, respectively. (Fig. 2G). The ROC curve suggested that RM-RM had good sensitivity and specificity and was better than other clinical parameters (Fig. 2H). These clinical parameters included sex, age, T stage, N stage, M stage and clinical stage. Finally, we carried out univariate regression and multivariate regression analyses. The results (Fig. 2I) showed that RM-RM was closely related to OS and was potentially the most meaningful independent predictor for BLCA. As shown in Fig. 2J and Additional file 1: Figure S2B, we found that the distribution of RM-RS was routinely consistent with the distribution of other clinical findings. We also found that as RM-RS increased, the expression of FASN, MAP2 and BMP6 increased, and that of GPC2, CNOT6L, GALNT12 and CARD10 decreased.

### Justification of the prognostic value of the RM-RM in two BLCA databases and real-world study

**Fig. 3:**
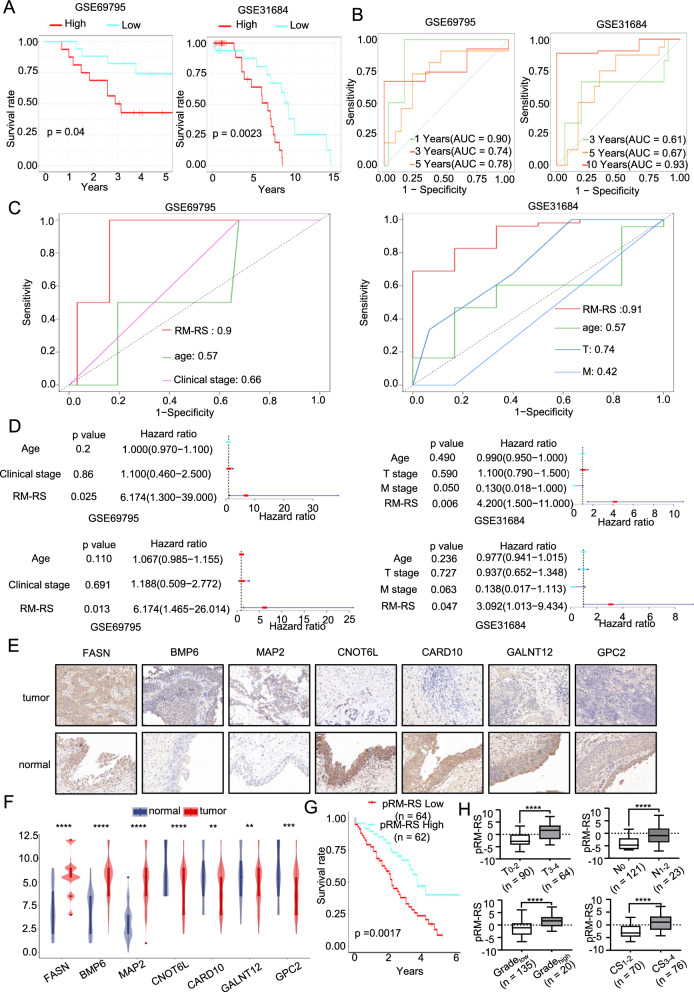
Justification of the Prognostic Value of the RM-RM in Two BLCA Databases and Real-World Study. **A** Kaplan–Meier analysis for overall survival (OS) curves of patients in low or high drug resistance and metabolism-related risk score (RM-RS) subgroups from two independent validation cohorts (GSE69795, GSE31684). **B** The receiver operating characteristic (ROC) curves of 1-, 3-, and 5 year OS of patients in GSE69795 and of 3-, 5-, and 10 year OS of patients in GSE31684 were predicted based on RM-RS. **(C)** The ROC curve of RM-RS was compared with that of only other clinical features in GSE69795 and GSE31684. **D** Univariate and multivariate Cox regression analyses of RM-RS and only other clinical features in GSE69795 and GSE31684. **E**, **F** Immunohistochemical (IHC) staining was used to detect the protein expression of metabolism-related differentially expressed genes (RM-DEGs) (FASN, MAP2, BMP6, GPC2, CNOT6L, GALNT12 and CARD10) in 60 normal tissues and 170 tumor tissues. The immunohistochemical staining immune response score (IRS) score was statistically analyzed and the violin diagram shows a representative image. **G**, **H** pRM-RS was obtained by IRS and RM-RM. The median pRM-RS was divided into a high-risk group and a low-risk group, and KM analysis and difference analysis of other clinical features between the two subgroups were performed. *p < 0.05; **p < 0.01; ***p < 0.001; ****p < 0.0001

In order to verify the prognostic value of the RM-RM, we checked two databases including OS data of BLCA patients: GSE69795 and GSE31684. According to the calculation formula of RM-RS obtained above, we also calculated the RM-RS of each patient, and divided the patients into high-hazard clusters and low-hazard clusters in accordance with the RM-RS (Additional file 1: Figure S3A). Similar to the TCGA dataset, we obtained the connection between the RM-RS and the survival rate. The results demonstrated a noteworthy difference between the two clusters (Fig. 3A), and the higher the result of the RM-RS, the worse the prognosis of the patients (Additional file 1: Figure S3B). As shown in Fig. 3B, RM-RM has excellent diagnostic value in both short- and long-term survival rates. For the two independent validation sets, RM-RM was also superior to the other only clinical features in terms of diagnostic sensitivity and specificity (Fig. 3D). Next, we carried out univariate regression and multivariate regression analyses in accordance with the two databases. Although the clinical data of the two validation sets are not as comprehensive as the TCGA database, RM-RM was still the best independent predictor of OS for only the existing clinical data within the two independent BLCA cohorts. In addition, as shown in Additional file 1: Figure S3C, we obtained coherent results compared to the TCGA database in two validation sets by analyzing the correlation among RM-RS, clinical characteristics and independent prognostic genes expression.

Finally, we further verified the prognostic value of the RM-RM by using samples of collected tissues in a real-world Study. As shown in Fig. 3E and Additional file 1: Figure S3D, immunohistochemistry (IHC) was finished to detect the expression of genes in RM-RM on the basis of protein expression (pRM-RS), and pRM-RS was obtained according to the immune response score of genes in RM-RM. The results were consistent with the TCGA database. FASN, MAP2 and BMP6 were highly expressed in bladder cancer, while GPC2, CNOT6L, GALNT12 and CARD10 were expressed at low levels in bladder cancer (Fig. 3F). According to pRM-RS, BLCA patients with survival data were divided into two subgroups. The KM survival analysis also directly revealed that the OS of the high-hazard cluster was notably shorter than that of the low-hazard cluster (Fig. 3G). shows that pRM-RS was closely related to grade, T stage, M stage and clinical stage.

In summary, we concluded that RM-RM had a high diagnostic value of prognosis for BLCA.

### The molecular function and mechanism of RM-RM in BLCA

**Fig. 4:**
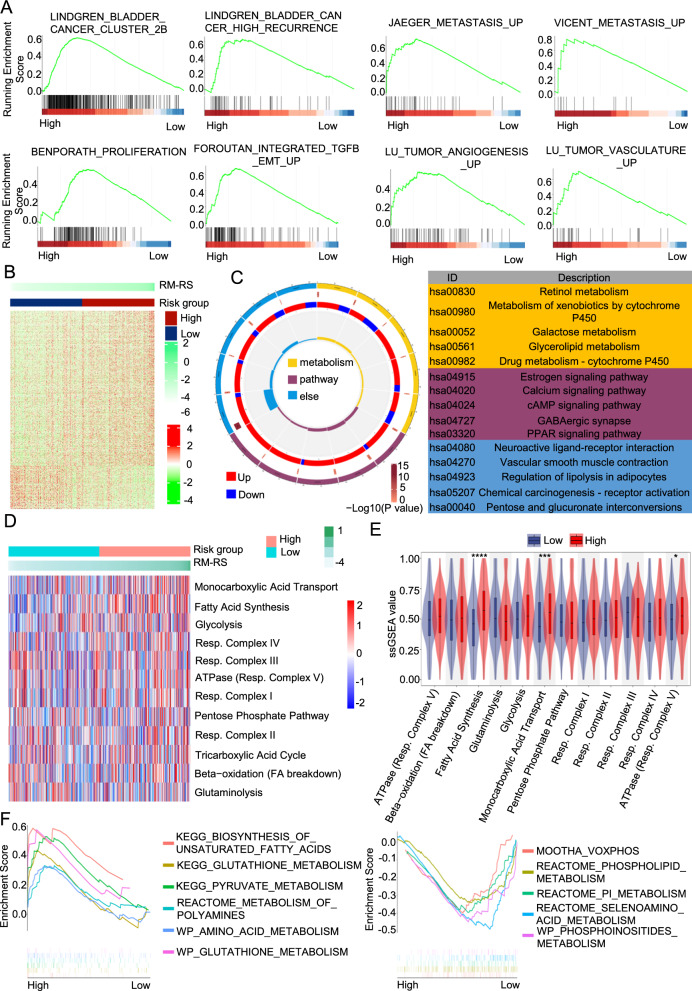
The Molecular Function and Mechanism of RM-RM in BLCA. **A** Gene set enrichment analysis (GSEA) of drug resistance and metabolism-related score (RM-RS) and BLCA occurrence, metastasis and progression signaling pathways. p < 0.05 **B** Heatmap of differentially expressed genes (DEGs) by comparing the expression between the high and low RM-RS groups. p < 0.05 and |FC|> 2. Blue represents the low-risk subgroup and red represents the high-risk subgroup. **C** Kyoto Encyclopedia of Genes and Genomes (KEGG) analysis of these DEGs. **D** Single-sample gene set enrichment analysis (ssGSEA) of metabolic pathway gene sets in the TCGA BLCA database. Heatmap of Metabolic pathway score of TCGA BLCA patients. **E** The violin plot shows the difference analysis of the metabolic scores of the high- and low-risk subgroups. **F** Metabolism-related gene sets enriched in the high- and low-risk subgroups (p < 0.05)

To analyze the molecular function of RM-RM, we completed GSEA and found that the risk model was strongly linked with the incidence, recurrence, distant metastasis, tumor proliferation and angiogenesis of BLCA **(**Fig. 4A**)**. With the purpose of further analyzing the mechanism of the model, we performed gene expression analysis on two risk subgroups and obtained 878 significantly differentially expressed genes (DEGs), of which 687 genes were overexpressed in the high-hazard subgroup and 191 genes were overexpressed in the low-hazard subgroup **(**Fig. 4B**)**. Through KEGG analysis, we discovered that the DEGs were strongly connected with drug metabolism, regulation of lipolysis in adipocytes, galactose metabolism and the PPAR signaling pathway (Fig. 4C). As shown in Additional file 1: Figure S4A, the result of GO analysis demonstrated that these DEGs were strongly linked with the reaction to the fibroblast growth factor, intermediate filament organization, cellular response to xenobiotic stimulus and intermediate filament cytoskeleton organization, suggesting that the two subgroups in RM-RM had different microenvironments and tumor stroma.

Energy metabolism is an important support for tumor function. Through ssGSEA, we obtained the metabolic score of each BLCA patient in the TCGA database **(**Fig. 4D**)**. As can be seen from the Fig. 4E and Additional file 1: Figure S4B, the high-hazard subclass was extensively higher than the low-hazard subclass in terms of fatty acid synthesis, monocarboxylic acid transport and ATPase (Resp. complex V). In the GSEA of representative metabolic pathways, we obtained the same results **(**Fig. 4F**)**. The results suggested that the high-hazard subgroup was mainly involved in fatty acid synthesis, while the low-hazard subgroup correlated with phosphoinositide metabolism. The two subgroups also showed different amino acid metabolism.

### RM-RM is correlated with the mutation and tumor microenvironment characteristics of BLCA

**Fig. 5:**
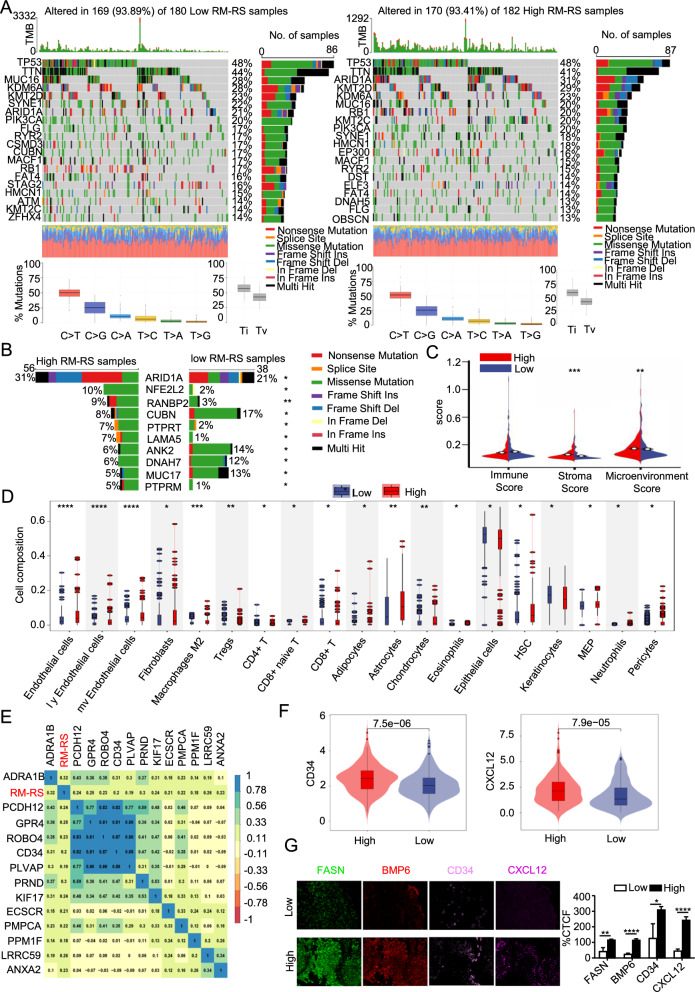
RM-RM is correlated with the mutation and tumor microenvironment characteristics of BLCA. **A** The top 20 mutated genes in different risk subgroups of TCGA BLCA were sorted according to the mutation rate. The color coding represents the mutation type. The total number of mutations is shown above, the percentage of mutations is shown on the right, and the distribution of base mutation types is shown below. **B** The top 10 genes with significant differences in mutant genes between the high- and low-risk subgroups. **C** Comparison of the immune, stromal and microenvironment scores in different risk subgroups. **D** Tumor microenvironment (TME) cells with significant differences in different RM-RS subgroups based on the x Cell algorithm. **E** Correlation heatmap of RM-RS and endothelial cell marker gene expression. **F** The expression of CD34 and CXCL12 in different risk subgroups. **G** Multiple immunofluorescence analysis (MIF) of different risk subgroups. The staining of these genes was quantified by corrected total cell fluorescence (CTCF). *p < 0.05; **p < 0.01; ***p < 0.001; ****p < 0.0001

Gene mutations can lead to the development of mutant cells which may have some selective advantages over adjacent cells. To explore the connection between gene mutations and drug resistance in BLCA, we analyzed gene mutations in the RM-RM subgroups, As can be seen from the Fig. 5A. By comparing the top twenty genes with the highest mutation rates, we suggested noteworthy variances in the gene mutation levels between the two groups. Missense variation was the most frequent category, and the results demonstrated no obvious differences compared to the transition and transversion of mutant genes between the two subgroups. Among the six transition and transversion events of the subgroups, the proportion of groups c and t demonstrated the highest transitions. By comparing the mutation probability of the two subgroups, we obtained the top ten most distinct differences of mutant genes **(**Fig. 5B**)**. These gene mutations may be an important factor leading to the progression of drug resistance in BLCA.

The tumor microenvironment (TME) mainly includes tumor cells, tumor extracellular matrix, immune cells, cancer-associated fibroblasts (CAFs), cancer-associated adipocytes and tumor-derived endothelial cells (TECs). The TME could be subdivided into an immunological microenvironment led by immune cells and a nonimmunological microenvironment led by cancer-associated fibroblasts. As shown in Fig. 5C, the stroma score of the high RM-RS cluster was higher than that of the low RM-RS cluster, suggesting that the nonimmunological microenvironment led by fibroblasts in the high-hazard cluster was more vigorous than the nonimmunological microenvironment led by fibroblasts in the low-hazard cluster. The detailed TME regulatory pathway of GSEA enriched by RM-RM mainly included positive regulation of fibroblast proliferation, responses to the drug, carcinoma-associated fibroblasts and angiogenesis, as shown in Additional file 1: Figure S5A. Then, we used three classical algorithms: xCell, MCP-counter and EPIC, to calculate the ratio of TME cells in BLCA patients from the TCGA database (Fig. 5D and Additional file 1: Figure S5B). We found that CAFs, endothelial cells, adipocytes and CD4^+^ T cells were more abundant in the high RM-RS subgroup. As shown in Fig. 5E, F and Additional file 1: Figure S5C, the RM-RS subgroup was strongly connected with the marker gene expression of TECs and CAFs. The higher the risk genes expression (FASN and BMP6) **(**Fig. 5G**)**, the higher the expression of an endothelial cell marker gene (CD34) and a fibroblast cell marker gene (CXCL12).

### Sensitivity of Drugs in the two RM-RS Subgroups

**Fig. 6:**
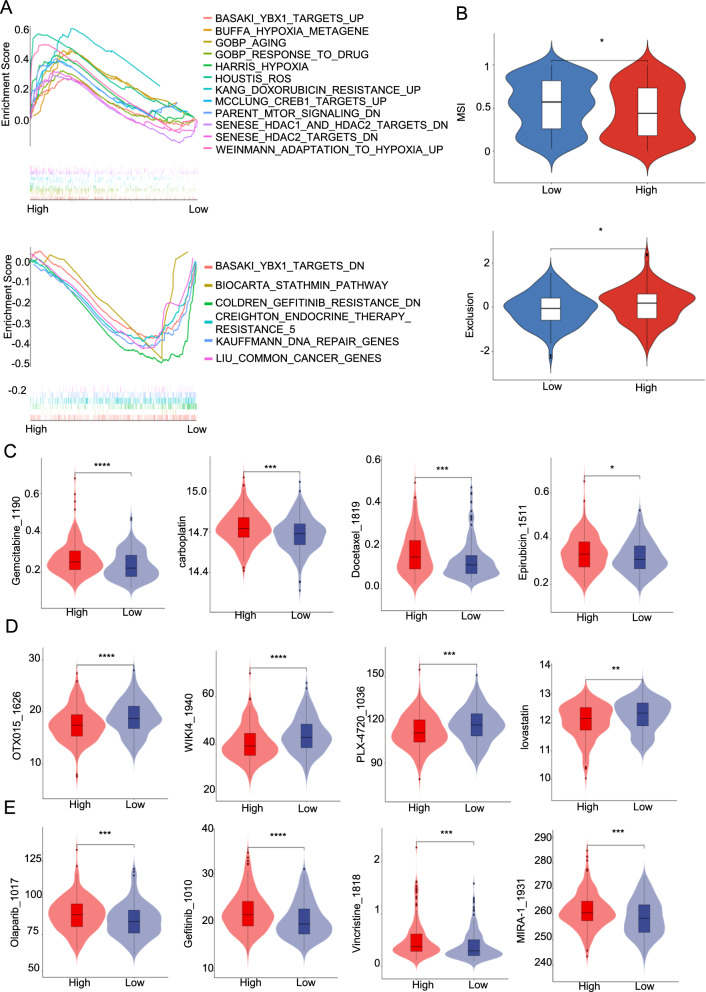
Sensitivity of Drugs in the two RM-RS Subgroups. **A** Drug metabolism-related gene sets enriched in the high- and low-risk subgroups (p < 0.05, false discovery rate (FDR) < 0.25). **B** Comparison of MSI score and exclusion score in different risk subgroups. **C** Sensitivity assessment of different risk subgroups for the current clinical preferred drug therapy. **D**, **E** Prediction of sensitive drugs recommended by different risk subgroups

After viewing the previous KEGG analysis **(**Fig. 4C**)** which demonstrated that RM-RS is involved in drug metabolism, we further studied the different sensitivities of BLCA individuals to drugs in different RM-RS subgroups. First and foremost, we comprehensively analyzed the pathways of drug metabolism relevant to BLCA resistance through GSEA in the two RM-RS groups (Fig. 6A). These results indicated that the high RM-RS cluster was correlated with drug response, aging, hypoxia, and doxorubicin resistance pathways, while the low RM-RS group correlated with endocrine therapy resistance, DNA repair, and decreased resistance to gefitinib. As shown in Fig. 6B, the MSI score of the high RM-RS cluster was meaningfully lower than that of the low RM-RS cluster, and the exclusion score was meaningfully higher than that of the low RM-RS cluster. The results suggested that the immune escape potential of the high RM-RS group was enhanced, and the effect of immunotherapy drugs is poor. From the Additional file 1: Figure S5D it is shown that there was no meaningful difference in dysfunction scores between the two subgroups. To provide treatment guidance for different BLCA clusters, we compared the sensitivity of two RM-RS clusters to various anticancer drugs. For chemotherapeutic drugs commonly used in BLCA, the drug sensitivity of high RM-RS individuals was significantly lower than that of low RM-RS individuals such as gemcitabine, carboplatin, docetaxel and epirubicin **(**Fig. 6C**)**. Then, we recommended sensitive drugs in different subgroups. The high-risk **(**Fig. 6D**)** subgroup was sensitive to BRD2/3/4 inhibitors (e.g., OTX015_1626), tankyrase inhibitors (e.g., WIKI4_1940), B-RafV600E inhibitors (e.g., PLX-4720_1036), and HMG-CoA reductase inhibitors (e.g., lovastatin), while the low-risk **(**Fig. 6E**)** subgroup was sensitive to PARP inhibitors (e.g., Olaparib_1017), tyrosine kinase inhibitors (e.g., Gefitinib_1010), vincristine, and maleimide analogs (e.g., MIRA-1_1931).

### Upregulation of FASN promotes drug resistance and poor prognosis in BLCA

**Fig. 7:**
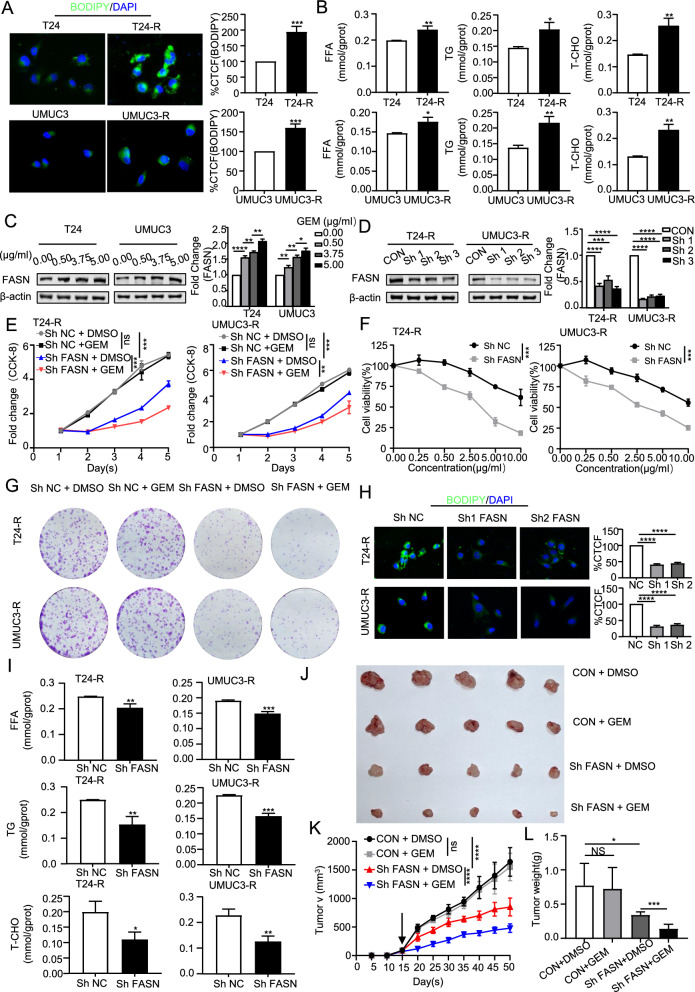
Upregulation of FASN promotes drug resistance and poor prognosis in BLCA. **A** The lipid content of bladder cancer cells and gemcitabine-resistant cells was quantified by BODIPY staining corrected total cell fluorescence (CTCF). **B** The contents of free fatty acids (FFAs), triglycerides (TGs) and total cholesterol (T-CHO) were used as intracellular lipid indexes. **C** The expression of FASN in bladder cancer cells resistant to different concentrations of gemcitabine was detected by Western blotting (WB). Density measurement and statistical analysis. Representative images are shown. **D** The expression of FASN in gemcitabine-resistant bladder cancer cells after FASN knockdown was detected by WB. **E**, **F** Cell viability and sensitivity to gemcitabine under all conditions were determined by CCK-8 assay. **G** The tumorigenic ability of single cells under all conditions was determined by colony formation assay. **H**, **I** The lipid content of gemcitabine-resistant cells after FASN knockdown was quantitatively detected by BODIPY staining corrected total cell fluorescence (CTCF) and the contents of free fatty acids (FFAs), triglycerides (TGs) and total bilirubin (T-CHO). **J**, **K**, and** L** Mice with stable knockdown expression of T24-R xenografts were treated with vector control or gemcitabine (50 mg/kg. IP. QOD) for approximately 5 weeks. Tumor volumes were measured every 5 days (n = 5 per group). Tumors were weighed after resection. The graphs show the means ± SEMs. One-way ANOVA followed by Tukey’s multiple comparison test. α = 0.05; *, p < 0.05; **, p < 0.01; ***, p < 0.001; ****p < 0.0001; ns, no significance

Through the analysis of the molecular function and mechanism of RM-RM in bladder cancer, we found that BLCA resistance is closely related to lipid metabolism. In order to further uncover the connection between genes in RM-RM and gemcitabine resistance in BLCA, we established another bladder cancer gemcitabine-resistant cell line (UMUC3). (Additional file 1: Figure S6A). The BODIPY assay **(**Fig. 7A**)** was conducted, and the results validated our prediction that lipid metabolism in drug-resistant cells is more active. We detected the content of free fatty acids (FFAs), triglycerides (TGs), and total cholesterol (T-CHO) in gemcitabine-resistant cells and normal BLCA cells **(**Fig. 7B**)**, and the results showed lipid accumulation in T24 gemcitabine-resistant (T24-R) cells and UMUC3 gemcitabine-resistant (UMUC3-R) cells. Through the establishment of the RM-RM, we found that FASN has the highest risk ratio **(**Fig. 2C**)**. As shown in Fig. 7C, we demonstrated that FASN is overexpressed in drug-resistant BLCA cells. In order to prove the function of FASN in the development of gemcitabine resistance, we first established T24 and UMUC3 BLCA gemcitabine-resistant cells with stable low expression of FASN **(**Fig. 7D**)**.

Subsequently, we used gemcitabine to treat T24-R and UMUC3-R cells with FASN knockdown. The results showed that T24-R and UMUC3-R cells were refractory to gemcitabine, and T24-R and UMUC3-R cells with FASN knockdown had restored sensitivity to gemcitabine, indicating that FASN promotes gemcitabine resistance in BLCA **(**Fig. 7E**)**. It also indicated that the overexpression of FASN promotes the proliferation of BLCA cells. The sensitivity of FASN knockdown on T24-R and UMUC3-R cells to gemcitabine was consistent with the above results **(**Fig. 7F**)**. In the Colony formation assay shown in Fig. 7G, after FASN expression was knocked down, the tumorigenic ability of single cells of drug-resistant cells was inhibited, and the inhibitory effect was more obvious under gemcitabine treatment, while the control group was not sensitive to gemcitabine (Additional file 1: Figure S6B). Through the BODIPY assay **(**Fig. 7H**)** and the determination of lipid content (Fig. 7I), we further verified the relationship between the expression of FASN and cellular lipid metabolism. The results showed that the intracellular lipid content decreased after FASN knockdown. Therefore, we can conclude that FASN further promotes drug resistance progression in BLCA cells by affecting lipid accumulation in BLCA cells. In order to further verify our prediction, we conducted in vivo tumor formation experiments in mice **(**Fig. 7J**)**. As shown in Fig. 7K, L, after FASN knockdown, the tumor growth rate was significantly repressed and the resistance of the tumor to gemcitabine was reversed. Altogether, the above results revealed that knockdown of FASN inhibit tumorigenesis of gemcitabine-resistant BLCA cells in vitro and in vivo.

### TVB-3166 inhibited BLCA progression and reversed gemcitabine resistance

**Fig. 8:**
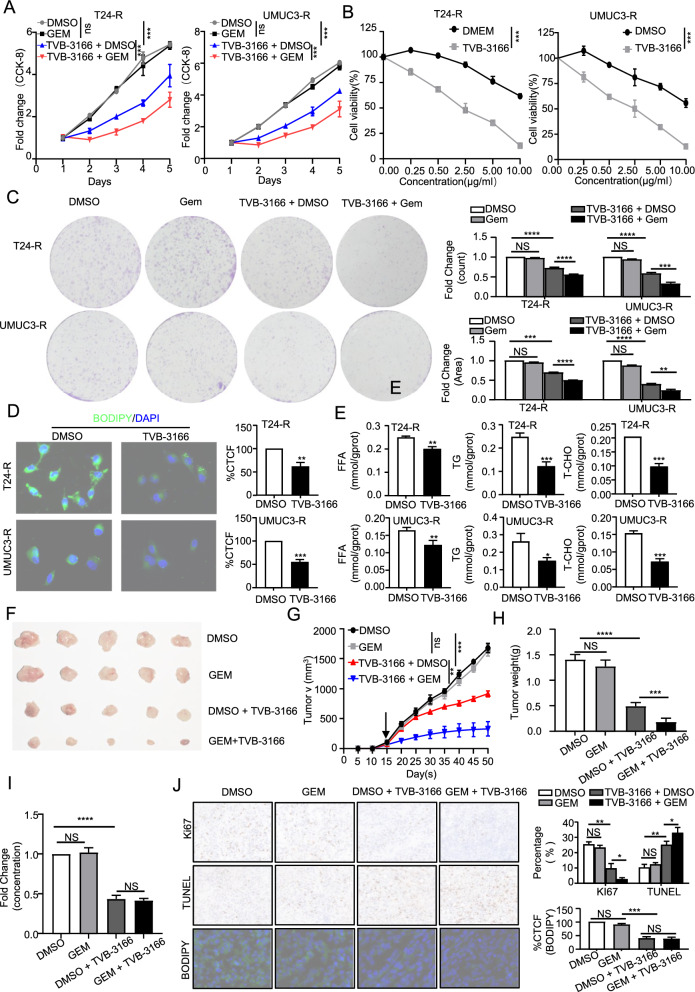
**| **TVB-3166 inhibited BLCA progression and reversed gemcitabine resistance. **A**, **B** Cell viability and sensitivity to gemcitabine under all conditions were determined by CCK-8 assay. **C** The tumorigenic ability of single cells under all conditions was determined by colony formation assay. **D**, **E** The lipid content of gemcitabine-resistant cells after treatment with TVB-3166 was quantitatively detected by BODIPY staining corrected total cell fluorescence (CTCF) and the contents of free fatty acids (FFAs), triglycerides (TGs) and total cholesterol (T-CHO). **F**, **G**, and **H** The mice were divided into 4 groups with 5 mice in each group: Group I (DMSO: DMSO), group II (DMSO: gemcitabine), group III (DMSO: TVB-3166) and group IV (gemcitabine: TVB-3166). Tumor volumes were measured every 5 days (n = 5 per group). Tumors were weighed after resection. The graphs show the means ± SEMs. One-way ANOVA followed by Tukey’s multiple comparison test. α = 0.05; *, p < 0.05; **, p < 0.01; ***, p < 0.001; ****p < 0.0001; ns, no significance. **I** Enzyme-linked immunosorbent assay (ELISA) was used to determine the FASN content of xenograft tumors in each group. **J** Statistical analysis was performed on the rate of KI67- and TUNEL-positive cells in each group of xenograft tumors by immunohistochemical staining (IHC). The corrected total cell fluorescence (CTCF) of BODIPY staining was used to quantitatively detect the lipid content of xenograft tumors in each group. Representative images are shown

TVB-3166 is an orally active, reversible and selective inhibitor of FASN. As shown in Additional file 1: Figure S6C, under the action of TVB-3166, the FASN content of T24-R and UMUC3-R cells was significantly reduced. The results of plate cloning experiments and CCK-8 assays indicated that TVB3166 could almost completely eliminate the influence of FASN on the proliferation and gemcitabine resistance of T24-R and UMUC3-R cells (Fig. 8A, B, C). also shows that TVB-3166 reversed gemcitabine resistance in BLCA. Second to the lipid changes presented after TVB-3166 treatment, as shown in the BODIPY assay **(**Fig. 8D**)** and the determination of lipid content **(**Fig. 8E**)**, the treatment group showed lower lipid aggregation than the contrast group. Next, in vivo experiments, we obtained consistent results **(**Fig. 8F–H**)**. These results demonstrated that compared with the control group, the volume, proliferation rate and mass of subcutaneous tumors treated with TVB-3166 decreased, while the volume, proliferation rate and mass of subcutaneous tumors treated with gemcitabine were not meaningfully different from those of the contrast group. The volume, proliferation rate and mass of subcutaneous tumors in mice treated with gemcitabine after TVB-3166 treatment significantly decreased, indicating that TVB-3166 improved the sensitivity to gemcitabine in gemcitabine-resistant BLCA cells. The ELISA results (Fig. 8I) showed that TVB-3166 reduced the FASN gene of the tumor, consistent with the in vitro results. BODIPY staining and IHC assay detection were carried out on subcutaneous tumors **(**Fig. 8J**)**. TVB-3166 treatment can reduce lipid accumulation, inhibit cell proliferation and increase the apoptosis rate. Thus, these results proved that by contrast with the control group, the proliferation and apoptosis rate of mice xenograft tumor cells treated with gemcitabine alone did not change significantly, while the proliferation rate of mice xenograft tumor cells treated with gemcitabine after TVB-3166 was inhibited and the apoptosis rate increased, indicating that TVB-3166 reversed gemcitabine resistance.

## Discussion

Gemcitabine is the most common drug in cancer chemotherapy, including BLCA. The occurrence of gemcitabine resistance remains the most important challenge in the treatment of tumor patients [15]. Drug-resistant cancers, under pharmacological pressure, exhibit complex molecular mechanisms aimed to inhibit treatment [16]. Gu J et al. found a novel therapeutic target to overcome gemcitabine resistance in pancreatic cancer [17]. Studies have found that cisplatin resistance in BLCA is related to epigenetic mechanisms such as DNA methylation, noncoding RNA regulation, m6A modification and posttranslational modification. Cocetta V et al. described the relationship between cisplatin resistance and cancer metabolism in detail [18]. However, there are a lack of systematic studies on gemcitabine resistance in BLCA cells. In our study, we identified and analyzed RM-DEGs based on RNA sequencing of gemcitabine-resistant BLCA cells and metabolic-related genes (MRGs). We also constructed and validated an RM-RM for predicting the OS of BLCA patients using several BLCA databases.

Tumor cell metabolism is a representative pattern of variable, alloplastic, and adaptive phenotypic characteristics. It is the result of a combination of internal and external factors that enable cancer cells to outlive, pervade the body, and obtain resistance to antineoplastic drugs [19]. Bacci M et al. described the function of abnormal lipid metabolism in affecting the antitumor treatment response and maintaining drug resistance [20]. Considering the essential role of tumor metabolism in chemotherapy resistance, we collected all MRGs on the basis of the MSigDB, established a gemcitabine-resistant cell line of BLCA cells, scientifically and thoroughly considered the metabolic model of BLCA resistance, and designed an RM-RM on the basis of OS to support precise prognosis information and guidance of treatment for BLCA patients.

In our research, we initially recognized and considered RM-DEGs in the TCGA BLCA dataset. The RM-DEGs were mostly associated with fatty acid and amino acid metabolism. Notably, these RM-DEGs were also enriched in extracellular matrix organization and drug metabolic processes. According to these RM-DEGs, we subdivided BLCA sufferers into two clusters with noteworthy variances in OS. These findings indicated the heterogeneity of BLCA metabolism, and that BLCA patients with diverse modes of metabolism have different prognoses.

Then, by means of univariate, LASSO and multivariate Cox regression analyses, we selected seven central RM-DEGs related to survival. Based on these seven genes, the TCGA dataset was viewed as the training data to create an RM-RM for predicting the survival of BLCA sufferers. The consequences showed that RM-RS was closely related to T, N, M and clinical stage, revealing that the deterioration of BLCA was associated with the reprogramming of tumor metabolism. Afterward, we used a variety of analytical methods to further prove that RM-RM was a reliable detached predictor and demonstrated the highest accuracy compared with other clinical indicators. Subsequently, two GEO datasets were used to further verify that RM-RM could be a promising clinical predictor for BLCA treatment. To discover the prospect of RM-RM for clinical conversion, we used immunohistochemistry to detect the protein expression levels of clinical specimens. The study further found that RM-RS in these patients was intimately linked with the prognosis and clinical characteristics.

The metabolism of BLCA patients represents a key issue for cancer research. Cao D et al. found that some genes, through inhibiting glucose metabolism, repressed tumor proliferation and improved cisplatin-induced apoptosis of BLCA cells [21]. We divided BLCA patients into two subclasses of different RM-RSs subgroups and performed GSEA and ssGSEA analysis. It was found that gemcitabine resistance in BLCA cells was closely related to lipid metabolism. Patients in the high RM-RS group showed more active lipid synthesis than those in the low RM-RS group.

Through gene mutation analysis, we uncovered considerable differences between the two different RM-RS subgroups. Previous studies have shown that ARID1A gene alterations may mediate resistance to platinum-based chemotherapy and estrogen receptor degradation/modulators [22]. Our study also found the top ten genes with the most obvious differential mutations, including ARID1A. The specific mechanisms of these genes are subjected to further research. In addition, current researches have proven that the TME plays a vital role in the procedure of tumor drug resistance [23] and have also proven the cross-link interference between metabolic reprogramming of cancer cells and the changes in the TME [24, 25]. Saw PE et al. proposed targeting cancer-associated fibroblasts (CAFs) to overcome anticancer drug resistance [26]. Particularly, in our study, we discovered that endothelial cells and fibroblasts obviously infiltrated in the TME of the high RM-RS subgroup. This may provide a new therapeutic target for patients with chemotherapy resistance of BLCA.

After that, we also found that the high RM-RS group was insensitive to a variety of classic chemotherapy regimens but was sensitive to other drugs, such as antiangiogenic drugs (B-RafV600E inhibitor: PLX-4720_1036) and lipid-lowering drugs (lovastatin). By predicting the different sensitivities of the two groups to anticarcinogen, we could precisely provide compatible drugs for patients with different metabolic sensitivities, suggesting the potential application of RM-RM in clinical guidance in the future.

The key genes in the RM-RM include FASN (Fatty Acid Synthase), MAP2 (Microtubule Associated Protein 2), BMP6 (Bone Morphogenetic Protein 6), GPC2 (Glypican 2), CNOT6L (CCR4-NOT Transcription Complex Subunit 6 Like), GALNT12 (Polypeptide N-Acetylgalactosaminyltransferase 12) and CARD10 (Caspase Recruitment Domain Family Member 10). We found that FASN, MAP2, and BMP6 were upregulated in BLCA tissues, while GPC2, CNOT6L, GALNT12 and CARD10 were downregulated. FASN is an essential enzyme in fatty acid synthesis [27]. It not only plays a vital role in lipometabolism, but also is related to tumor proliferation. In addition, FASN can adjust the immune microenvironment and take part in epithelial-mesenchymal transition, thereby regulating tumor progression [28]. Li Y et al. found that FASN was associated with sorafenib resistance in patients with liver cancer [29]. MAP2 belongs to the microtubule-associated protein of the MAP2/Tau family, which is related to the collection of signal proteins and the modulation of microtubule-mediated transport [30]. Pulkkinen HH et al. found that BMP protein regulates angiogenesis and endothelial cell proliferation [31]. GPC2 protein is a promising therapeutic target for pantumor [32]. Katsumura S et al. found that CNOT6L protein can coordinate energy intake and consumption when stimulated [33]. Guda K et al. identified the mutation of GALNT12 protein in colon cancer patients and explored its function in the occurrence and progression of colon cancer [34]. CARD10 protein mediates the occurrence and progression of various kinds of cancers [35]. Zhu L et al. revealed that CARD10 protein also plays a crucial role in the formation of a growth factor signaling axis that mediates immunosuppression and tumorigenesis by TBKBP1 and TBK1 [36].

FASN, as a representative gene, was further verified as a promoting factor for gemcitabine resistance in vitro and in vivo. Previous researches have proven that the effect of a FASN inhibitor (TVB-3166) on carcinogenic signals and gene expression enhances the antitumor efficacy of various xenograft tumor models [37]. Our study further demonstrated that TVB-3166 can reverse gemcitabine resistance.

In summary, this study constructed an RM-RM with high diagnostic accuracy for predicting OS and treatment response in patients with bladder cancer. We hope that the constructed RM-RM can provide guidance in the treatment of BLCA patients.

## Materials and methods

### Cell culture and reagents

T24 and UMUC3 cells were obtained from the American Type Culture Collection (ATCC, Manassas, VA). These BLCA cells were cultured in DMEM (for UMUC3 and UMUC3-R) and RPMI-1640 (for T24 and T24-R) mediums added into 10% foetal bovine serum (Gibco, USA). The lentivirus was used to knock down FASN and the vector were purchased from GeneChem. Our operation steps were strictly used for maintaining the instruction requirements. Gemcitabine (HY-17026) and TVB-3166 were purchased from MCE.

### Establishment of gemcitabine-resistant cell lines

Two types of BLCA cell lines (T24, UMUC3) were first incubated with gemcitabine in several concentration gradients (0–20 μg/ml) for 48 h. The IC50s were calculated depending on their absorbances. Then, the cell lines were cocultured with gemcitabine at the concentration levels at which the IC50s were attached. Repeat the above steps. RI (resistance index) is calculated as the IC50 of the drug-resistant cells divided by the IC50 of the wild-type (WT) cells. 1–5 indicated low drug resistance, 5–15 indicated moderate drug resistance, and more than 15 indicated high drug resistance. Previous studies have found that the IC50 values of gemcitabine-resistant cells in two cell lines are 5 to 10 times higher or more compared to WT cells [38]. When the RI (resistance index) > 5, then we considered that drug-resistant cell lines were successfully constructed.

### RNA sequencing

Three groups of repeated cells were used for RNA sequencing after the establishment of the T24 gemcitabine-resistant cell line. This sequencing was completed by APT (APPLIED PROTEIN TECHNOLOGY). By means of the R “limma” (Version 3.54.0) package [39], we checked out the drug-resistant differential genes of gemcitabine resistance (p < 0.05, |Fold change|> 2).

### Data acquisition

The MRGs were collected from MSigDB [40]. The TCGA BLCA database was acquired from UCSC Xena as the training set, and two BLCA data: GSE69795 [41] and GSE31684 [42] were acquired from GEO as the validation set. The marker genes of endothelial cells and fibroblasts were collected from the literature, CellMarker database and R “xCell” (Version1.1.0) package [43].

### Visualization of differentially expressed genes

The volcano plot and heatmap are presented by R “ggplot2” (Version 3.4.0) [44] to demonstrate the distribution of DEGs. Moreover, the Venn diagram demonstrated the connection of differentially expressed genes of gemcitabine resistance (R-DEGs) and metabolic-related genes (MRGs) to gain resistance and metabolism-related differentially expressed genes (RM-DEGs).

### Enrichment analysis of genes

GO analysis and KEGG analysis were carried out by the R “clusterProfiler” (Version 4.6.0) package to deeply study the major molecular functions and significantly enriched pathways of the DEGs [45]. We took P < 0.05 as the standard of significant difference.

### Unsupervised clustering analysis

We used the R ConsensusClusterPlus' (version 1.62.0) package to perform hierarchical consistency clustering analysis [46].

### Establishment and validation of the drug resistance and metabolism-related prognosis risk assessment model (RM-RM)

First, we screened out the main genes that correlated with the OS of BLCA patients from RM-DEGs by using univariate Cox regression. Then, the R “glmnet” (Version 4.1–6) package [47] was used for LASSO Cox regression to evade the overfitting of characteristics and to narrow the number of factors for predicting OS. Finally, we further evaluated the genes that were recognized by LASSO regression using multiple Cox regression analysis, obtained seven key genes, and used them to create a forecast risk model on the basis of drug resistance and metabolism. The drug resistance and metabolism-related risk score (RM-RS) formula is described below: RM-RS = ∑ (β × Exp), in which β and Exp are respectively representation of coefficient and genes expression that were standardized.

### Survival analysis

In accordance with the median RM-RS, patients with BLCA were subdivided into a high RM-RS group and a low RM-RS group. KM survival analysis was used to prove the variance in OS of different RM-RS groups. In addition, the ROC curves were used to estimate the prognostic value of the RS-RS using the R “Survival ROC” package. Subsequently, through the R “survival” (Version 3.5–5) package, we carried out the analysis of independent factors affecting BLCA prognosis by univariate and multivariate regression. The above determination methods were verified in two independent gene sets.

### Immunohistochemical staining assay

We performed an immunohistochemical staining assay on the tissue chips in accordance with a previously described method [48]. The antibodies we used included anti-GPC2 (1:200, AF2304SP, Goat, IgG, Novus), anti-CNOT6L (1:75, abs108959, Rabbit, IgG Absin), anti-FASN (1:300, 66,591-1-Ig, Mouse, IgG, Proteintech), anti- MAP2 (1:2500, 66,846-1-Ig, Rabbit, IgG, Proteintech), anti-BMP6 (1:500, bs-10090R, Rabbit, IgG, Bioss), anti-CARD10 (1:300, bs-7081R, Rabbit, IgG, Bioss), anti-GALNT12 (1:100, ab201196, Rabbit, IgG, Abcam), anti-IgG (ab238004, Mouse, Abcam), anti-IgG (A7007, Goat, Beyotime), and anti-IgG (30,000-0-AP, Rabbit, Proteintech). The IRS (value, 0–12) was calculated by multiplying the staining strength grade by the positive area grade. The grade of staining strength was prescribed below: 0, negative; l, weak; 2, moderate; and 3, strong. The positive area grade was described as follows: zero-grade, less than 5%; first-grade, 5% to 25%; second-grade, 26% to 50%; third-grade, 51% to 75%; and fourth-grade, greater than 75%.

### GSEA and ssGSEA

GSEA (Gene Set Enrichment Analysis) was carried out by means of the “clusterProfiler” package and GSEA software (4.3.2) to reveal the relevant signaling pathways, and visualization was implemented by means of the R “enrichplot” package (Version 1.20.0) and the “ggplot2” package. The ssGSEA (single sample Gene Set Enrichment Analysis) was performed by the R “GSVA” package (Version 1.48.3), and the individual score of each sample-specific pathway was obtained by the sample-related gene expression. In order to explore the metabolic pathway of BLCA gene sets, we searched for relatable papers [49, 50] and data in the MSigDB.

### Gene mutation analysis

We obtained somatic mutation information using the TCGA BLCA database. Meanwhile, using the R “Maftools” (Version 2.14.0) package, we analyzed various differences in mutations in the two RM-RS subgroups [51].

### Analysis of the TME

In order to evaluate the immune and stromal scores of each BLCA patient, we used different algorithms on the online tools: the xCell, MCP-counter, and EPIC. Subsequently, we evaluated the infiltration of different cells in the two subgroups by box plot visualization. Finally, we performed an association analysis by means of the R “corrplot” package (Version 0.92) to prove the intimate connection between RM-RS and characteristic cell marker genes.

### Multiplex immunofluorescence staining assay

We performed an immunofluorescence staining assay on the tissue in accordance with the method illustrated previously [52]. The antibodies we used included anti-FASN (1:200, 66,591-1-Ig, ProteinTech), anti-BMP6 (1: 3,000, bs-10090R, Bioss), anti-CXCL12 (1:200, 17,402-1-AP, ProteinTech) and anti-CD34 (1:200, ab81219, Abcam). Through ImageJ software analysis, we obtained corrected total cell fluorescence (CTCF) to evaluate the content of protein expression in BLCA and adjacent tissues.

### Prediction of drug sensitivity

With the purpose of predicting the sensitivity of two risk subgroups to multiple chemotherapeutic drugs, we jointly analyzed the TCGA database, Genomics of Drug Sensitivity in Cancer (GDSC) and Cancer Therapeutics Response Portal (CTRP) data. By means of the R “oncoPredict” package [53], we obtained the IC50 of each sample in two different RM-RS groups for hundreds of drugs.

### Statistical analyses

Our data processing was performed by R software (version 4. 2. 1).

### Human samples

With the agreement of the Ethics Committee of the First Affiliated Hospital of Zhengzhou University, we gathered BLCA tissues and normal bladder tissues from BLCA patients, partially collected them in a -80 °C freezer and partially embedded them in paraffin.

### BODIPY staining

First, we placed cells or fresh tissues in 4% paraformaldehyde solution. Subsequently, we incubated cells or tissues with BODIPY and DAPI in the dark for 30 min and 10 min. Then ImageJ software was used for analysis.

### FASN, FFA, TG and T-CHO measurement assay

The levels of FASN were assessed by enzyme-linked immunosorbent assay (ELISA) in accordance with the FASN ELISA kit’s instructions (Abcam, ab279412). The contents of FFAs, TGs and T-CHO were correspondingly assessed by an FFA assay kit, TG assay kit, and T-CHO assay kit (Nanjing Jiancheng Bioengineerin), in accordance with the instructions.

### Western blotting

The proteins of cells and tissues were extracted using RIPA buffer containing phosphatase and protease inhibitors. Subsequently, 30 µg of protein was put into a Bis–Tris gel to accomplish protein electrophoresis. Then, we transferred the proteins to polyvinylidene fluoride (PVDF) membranes and blocked the membranes in 5% skim milk. After that, we took the membrane together with primary antibodies overnight, incubated it with the second antibody for 1 h, and exposed the membrane. The antibodies included anti-FASN (1:1,000, 66,591-1-Ig, ProteinTech) and anti-β-actin (1:10,000, 20,536-1-AP, Proteintech).

### Cell proliferation assays

Gemcitabine-resistant cell lines (T-24 and UMUC3 cells) treated with TVB-3166 (1 μmol) or transfected with shRNA were treated with gemcitabine (5 μg/ml). Cell viability was determined by using Cell Counting Kit-8 (CCK-8) in accordance with the manufacturer's instructions [54].

### Drug sensitivity test

After transfection with shRNA for 48 h or treatment with TVB-3166 (1 μmol), gemcitabine-resistant cell lines (T-24 and UMUC3 cells) were treated with gemcitabine for 24 h at six concentrations (1 μg, 2 μg, 4 μg, 8 μg, 16 μg and 32 μg per ml). Their viabilities were detected by CCK-8 according to the guidelines provided by the manufacturer.

### Colony formation assay

Different reagents were added as needed: gemcitabine (5 μg/ml) and TVB-3166 (1 μmol). A total of 1000 cells were cultured per well of the 6-well plate for 1 week, followed by colony analysis.

### Supplementary Information

**Additional file 1:**Figure S1-6 and the corresponding legends.

**Additional file 2: **The list of OS-related RM-DEGs.

## Data Availability

All data are available in a public, open access repository. R and other custom scripts for analyzing data are available upon reasonable request.

## Declarations

### Animal experiments

The subcutaneous xenograft model was agreed by the Ethics Committee of Experimental Animal Center of Zhengzhou University. Male BALB/c nude mice (approximately 4 weeks old), purchased from Beijing Weitong Lihua Experimental Animal Technology, were divided into 8 groups with 5 mice per subgroup. Four groups be used for studying the effect of FASN knockdown on the reversal of gemcitabine resistance induced in vivo: Group I (DMSO: DMSO), group II (DMSO: gemcitabine), group III (FASN knockdown: DMSO) and group IV (FASN knockdown: gemcitabine). Four groups be used for studying the effect of TVB-3166 on the reversal of gemcitabine resistance in vivo: Group I (DMSO: DMSO), group II (DMSO: gemcitabine), group III (DMSO: TVB-3166) and group IV (gemcitabine: TVB-3166). In this experiment, 2*10^6^ BLCA cells were injected subcutaneously into each mouse. After that, the volume of the tumor was recorded every 5 days. Drug therapy was initiated when the average tumor size was measured at 100–200 mm^3^.After 50 days, we removed the subcutaneous tumor from the mice. After measurement and recording, we partially stored these tumors at in a − 80 °C freezer and partially embedded them in paraffin. Mice treated with gemcitabine were intraperitoneally injected with gemcitabine 50 mg/kg every 2 days. Mice treated with TVB-3166 were intragastrically administered 60 mg/kg TVB-3166 daily for approximately 5 weeks.
