# Supplementary material for: Metabolic reprogramming based on RNA sequencing of gemcitabine-resistant cells reveals the FASN gene as a therapeutic for bladder cancer
Source: J Transl Med. 2024 Jan 13;22:55. doi: 10.1186/s12967-024-04867-8 (PMC10787972; doi:10.1186/s12967-024-04867-8)
Supplement: Supplementary file 1 — Additional file 1:Figure S1-6 and the corresponding legends. [file 12967_2024_4867_MOESM1_ESM.pdf]

Sfig. 1

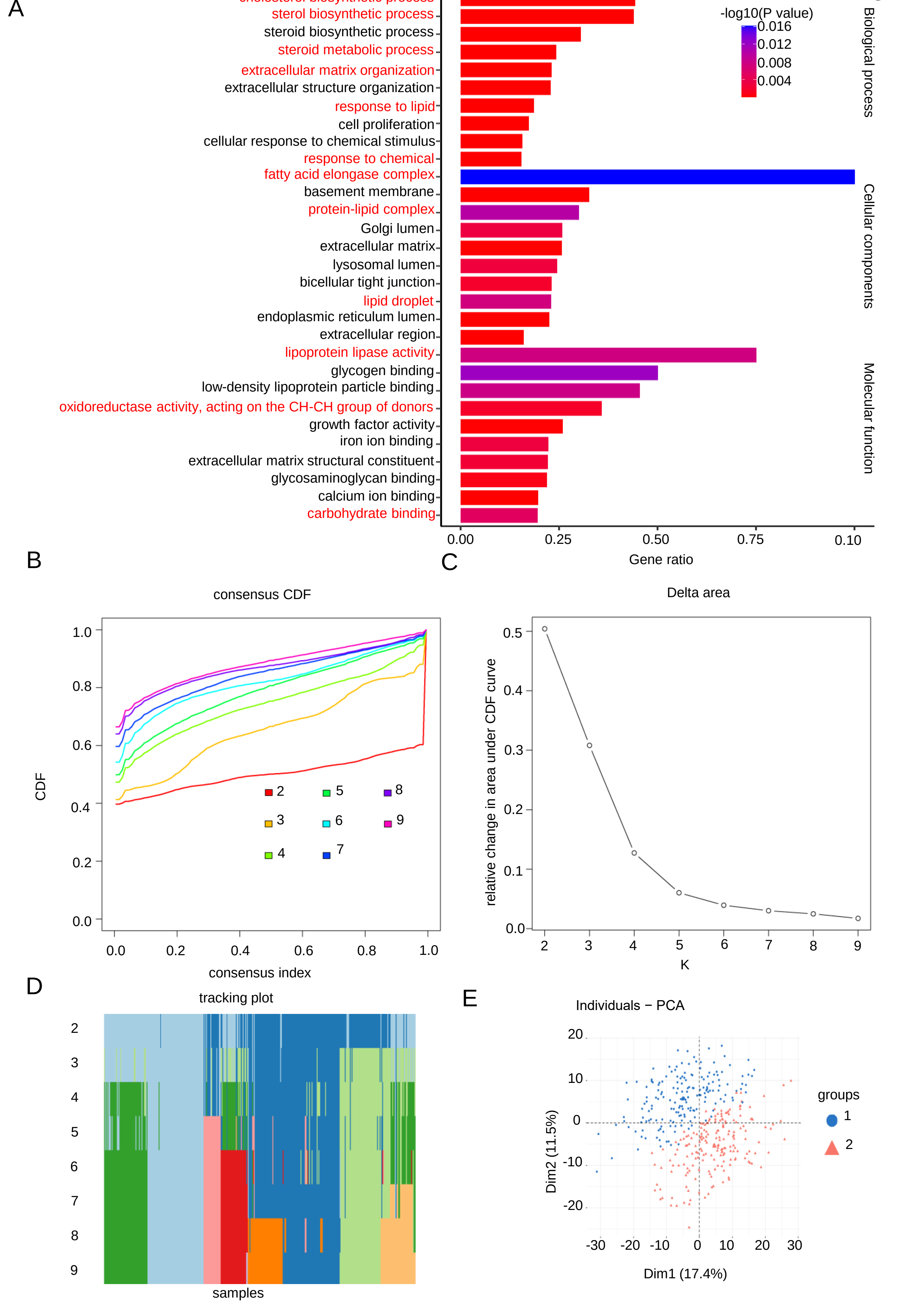

A
cholesterol biosynthetic process
sterol biosynthetic process
steroid biosynthetic process
steroid metabolic process
extracellular matrix organization
extracellular structure organization
response to lipid
cell proliferation
cellular response to chemical stimulus
response to chemical
fatty acid elongase complex
basement membrane
protein-lipid complex
Golgi lumen
extracellular matrix
lysosomal lumen
bicellular tight junction
lipid droplet
endoplasmic reticulum lumen
extracellular region
lipoprotein lipase activity
glycogen binding
low-density lipoprotein particle binding
oxidoreductase activity, acting on the CH-CH group of donors
growth factor activity
iron ion binding
extracellular matrix structural constituent
glycosaminoglycan binding
calcium ion binding
carbohydrate binding
-log10(P value)
0.016
0.012
0.008
0.004
Biological process
Cellular components
Molecular function
0.00
0.25
0.50
0.75
0.10
Gene ratio
B
consensus CDF
CDF
1.0
0.8
0.6
0.4
0.2
0.0
2
3
4
5
6
7
8
9
consensus index
C
Delta area
relative change in area under CDF curve
0.5
0.4
0.3
0.2
0.1
0.0
K
D
tracking plot
2
3
4
5
6
7
8
9
samples
E
Individuals − PCA
20
10
0
-10
-20
Dim2 (11.5%)
-30
-20
-10
0
10
20
30
Dim1 (17.4%)
groups
1
2

Sfig. 2

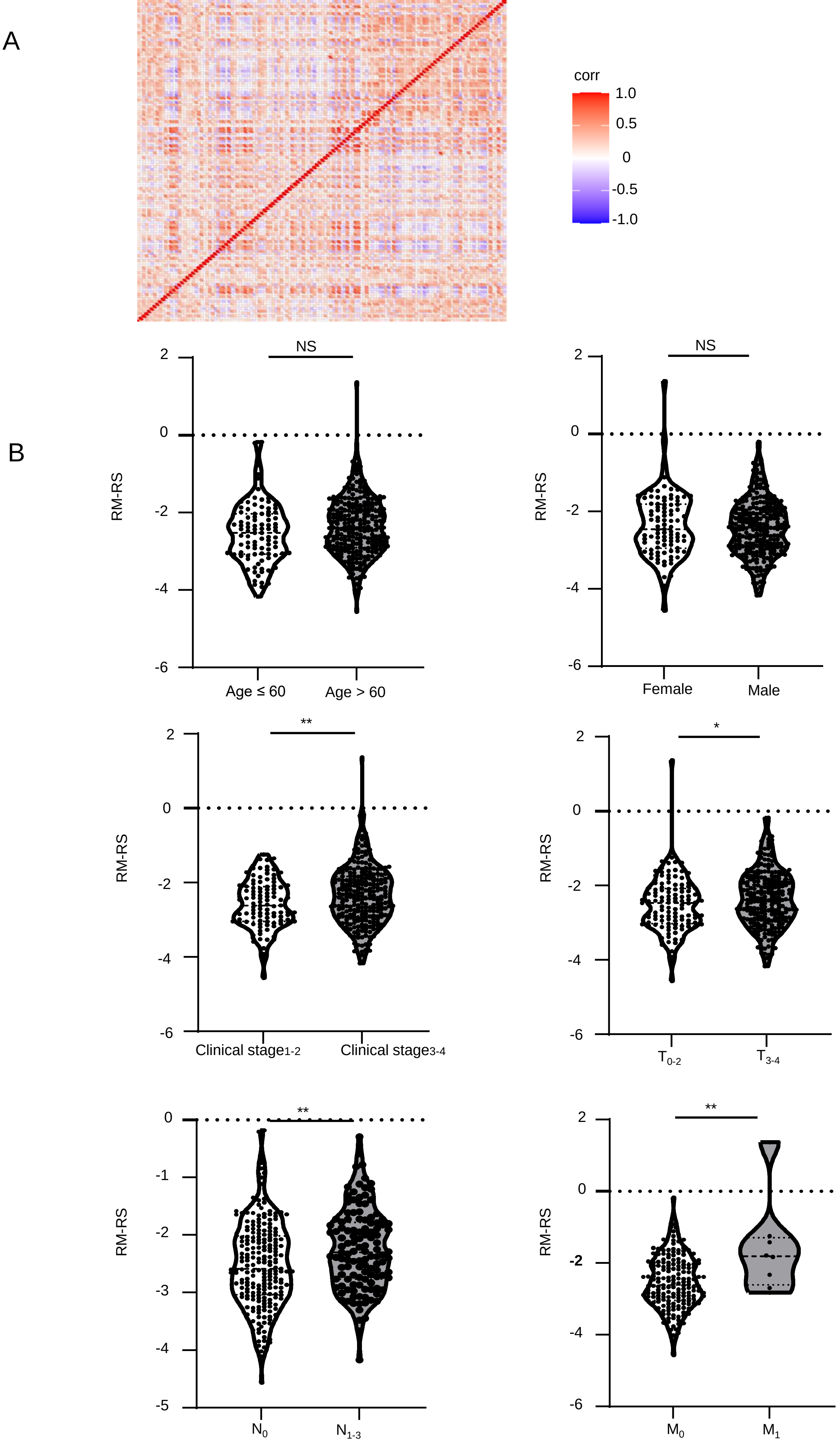

A
corr
1.0
0.5
0
-0.5
-1.0
B
RM-RS
NS
Age ≤ 60
Age > 60
NS
Female
Male
**
Clinical stage1-2
Clinical stage3-4
*
T0-2
T3-4
**
N0
N1-3
**
M0
M1

Sfig. 3

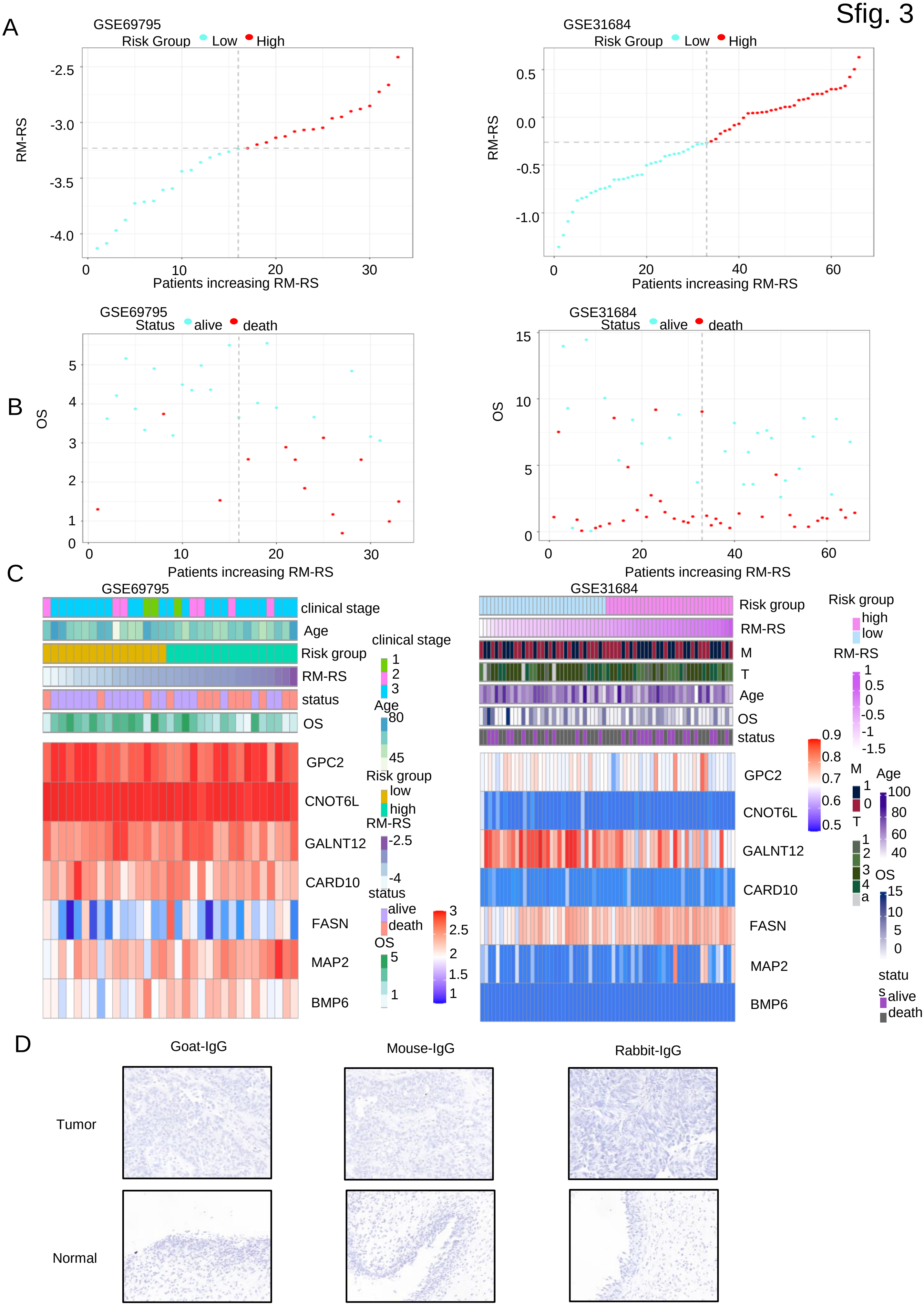

A
GSE69795
Risk Group Low High
RM-RS
Patients increasing RM-RS
GSE31684
Risk Group Low High
B
GSE69795
Status alive death
OS
GSE31684
Status alive death
C
GSE69795
clinical stage
Age
Risk group
RM-RS
status
OS
GPC2
CNOT6L
GALNT12
CARD10
FASN
MAP2
BMP6
low
high
alive
death
GSE31684
Risk group
RM-RS
M
T
Age
OS
status
D
Goat-IgG
Mouse-IgG
Rabbit-IgG
Tumor
Normal

Sfig. 4

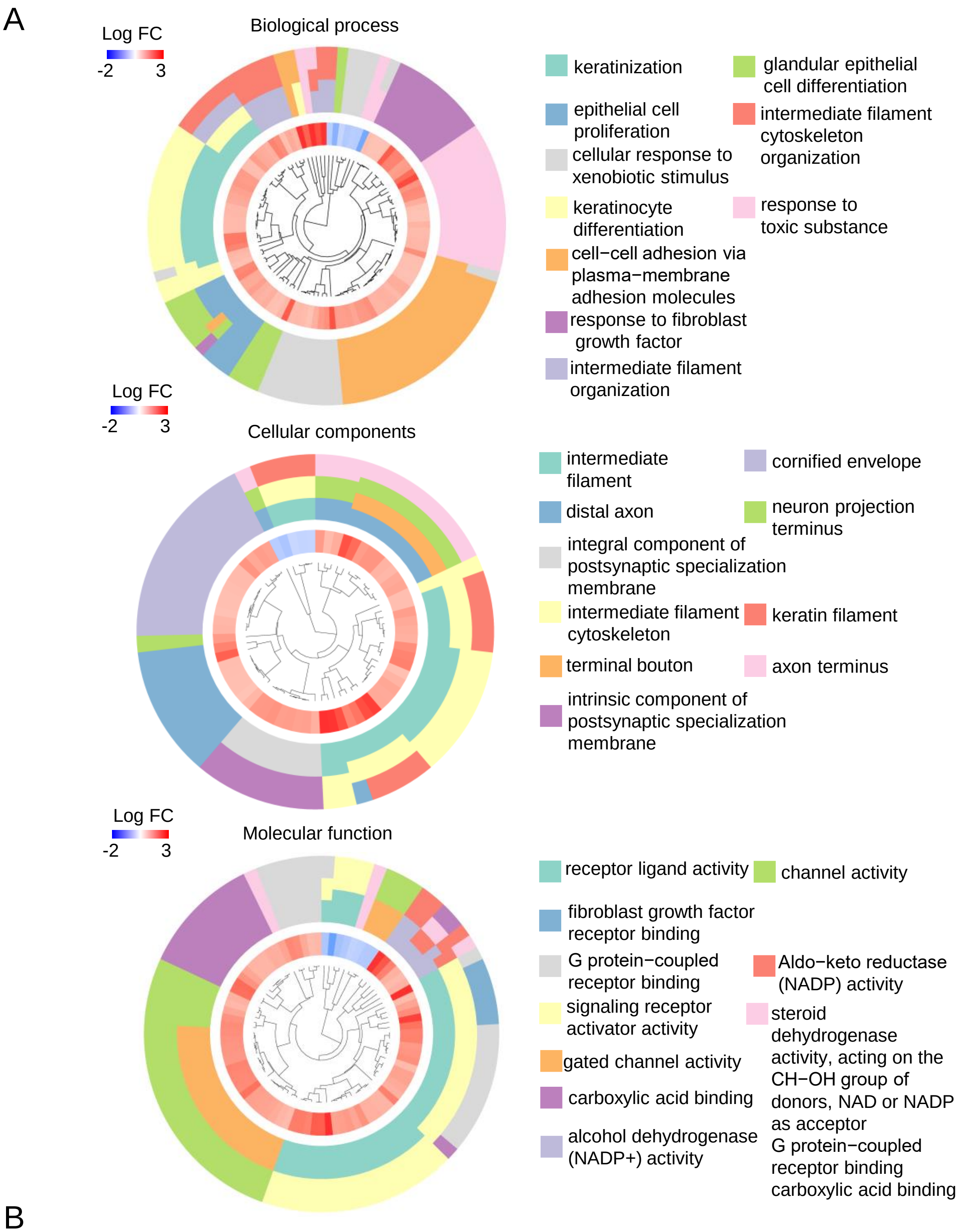
A
Log FC
-2
3
Biological process
keratinization
glandular epithelial cell differentiation
epithelial cell proliferation
intermediate filament cytoskeleton organization
cellular response to xenobiotic stimulus
keratinocyte differentiation
response to toxic substance
cell−cell adhesion via plasma−membrane adhesion molecules
response to fibroblast growth factor
intermediate filament organization
Log FC
-2
3
Cellular components
intermediate filament
cornified envelope
distal axon
neuron projection terminus
integral component of postsynaptic specialization membrane
intermediate filament cytoskeleton
keratin filament
terminal bouton
axon terminus
intrinsic component of postsynaptic specialization membrane
Log FC
-2
3
Molecular function
receptor ligand activity
channel activity
fibroblast growth factor receptor binding
G protein−coupled receptor binding
Aldo−keto reductase (NADP) activity
signaling receptor activator activity
steroid dehydrogenase activity, acting on the CH−OH group of donors, NAD or NADP as acceptor
gated channel activity
G protein−coupled receptor binding
carboxylic acid binding
carboxylic acid binding
alcohol dehydrogenase (NADP+) activity

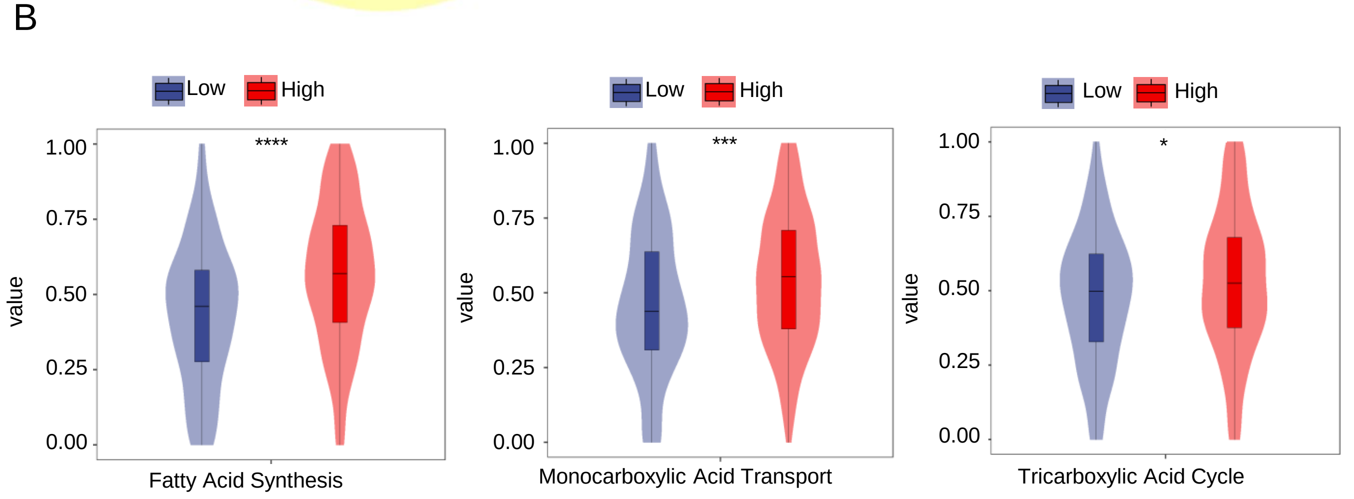
B
Low
High
value
1.00
0.75
0.50
0.25
0.00
****
Fatty Acid Synthesis
***
Monocarboxylic Acid Transport
*
Tricarboxylic Acid Cycle

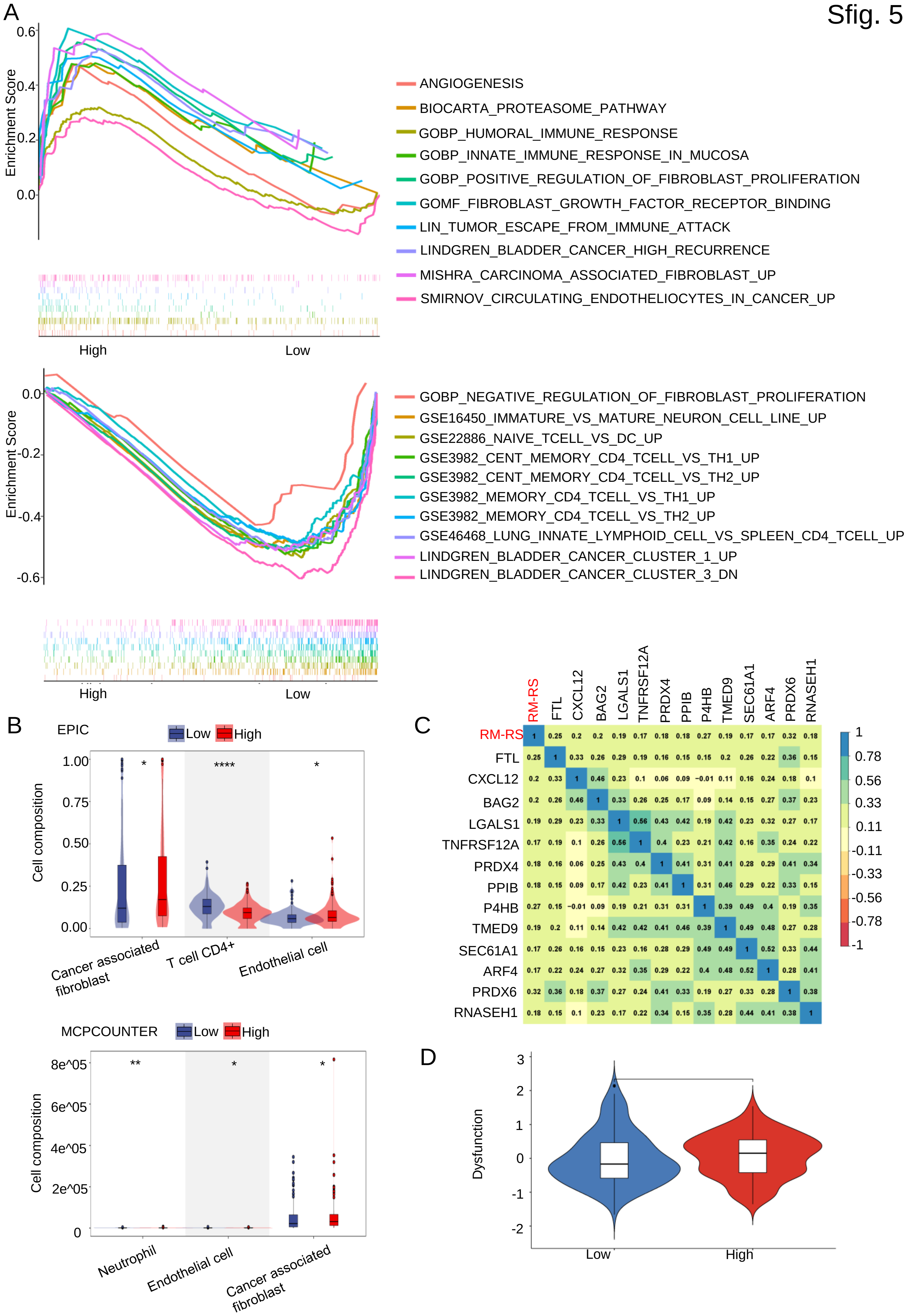
Sfig. 5
A
Enrichment Score
0.6
0.4
0.2
0.0
High
Low
ANGIOGENESIS
BIOCARTA_PROTEASOME_PATHWAY
GOBP_HUMORAL_IMMUNE_RESPONSE
GOBP_INNATE_IMMUNE_RESPONSE_IN_MUCOSA
GOBP_POSITIVE_REGULATION_OF_FIBROBLAST_PROLIFERATION
GOMF_FIBROBLAST_GROWTH_FACTOR_RECEPTOR_BINDING
LIN_TUMOR_ESCAPE_FROM_IMMUNE_ATTACK
LINDGREN_BLADDER_CANCER_HIGH_RECURRENCE
MISHRA_CARCINOMA_ASSOCIATED_FIBROBLAST_UP
SMIRNOV_CIRCULATING_ENDOTHELIOCYTES_IN_CANCER_UP
Enrichment Score
0.0
-0.2
-0.4
-0.6
High
Low
GOBP_NEGATIVE_REGULATION_OF_FIBROBLAST_PROLIFERATION
GSE16450_IMMATURE_VS_MATURE_NEURON_CELL_LINE_UP
GSE22886_NAIVE_TCELL_VS_DC_UP
GSE3982_CENT_MEMORY_CD4_TCELL_VS_TH1_UP
GSE3982_CENT_MEMORY_CD4_TCELL_VS_TH2_UP
GSE3982_MEMORY_CD4_TCELL_VS_TH1_UP
GSE3982_MEMORY_CD4_TCELL_VS_TH2_UP
GSE46468_LUNG_INNATE_LYMPHOID_CELL_VS_SPLEEN_CD4_TCELL_UP
LINDGREN_BLADDER_CANCER_CLUSTER_1_UP
LINDGREN_BLADDER_CANCER_CLUSTER_3_DN
B
EPIC
Low
High
Cell composition
1.00
0.75
0.50
0.25
0.00
*
****
*
Cancer associated fibroblast
T cell CD4+
Endothelial cell
MCPCOUNTER
Low
High
Cell composition
8e^05
6e^05
4e^05
2e^05
0
**
*
*
Neutrophil
Endothelial cell
Cancer associated fibroblast
C
RM-RS FTL CXCL12 BAG2 LGALS1 TNFRSF12A PRDX4 PPIB P4HB TMED9 SEC61A1 ARF4 PRDX6 RNASEH1
RM-RS 1 0.25 0.2 0.2 0.19 0.17 0.18 0.18 0.27 0.19 0.17 0.17 0.32 0.18
FTL 0.25 1 0.33 0.26 0.29 0.19 0.16 0.15 0.15 0.2 0.26 0.22 0.36 0.15
CXCL12 0.2 0.33 1 0.46 0.23 0.1 0.06 0.09 −0.01 0.11 0.16 0.24 0.18 0.1
BAG2 0.2 0.26 0.46 1 0.33 0.26 0.25 0.17 0.09 0.14 0.15 0.27 0.37 0.23
LGALS1 0.19 0.29 0.23 0.33 1 0.56 0.43 0.42 0.19 0.42 0.23 0.32 0.27 0.17
TNFRSF12A 0.17 0.19 0.1 0.26 0.56 1 0.4 0.23 0.21 0.42 0.16 0.35 0.24 0.22
PRDX4 0.18 0.16 0.06 0.25 0.43 0.4 1 0.41 0.31 0.41 0.28 0.29 0.41 0.34
PPIB 0.18 0.15 0.09 0.17 0.42 0.23 0.41 1 0.31 0.46 0.29 0.22 0.33 0.15
P4HB 0.27 0.15 −0.01 0.09 0.19 0.21 0.31 0.31 1 0.39 0.49 0.4 0.19 0.35
TMED9 0.19 0.2 0.11 0.14 0.42 0.42 0.41 0.46 0.39 1 0.49 0.48 0.27 0.28
SEC61A1 0.17 0.26 0.16 0.15 0.23 0.16 0.28 0.29 0.49 0.49 1 0.52 0.33 0.44
ARF4 0.17 0.22 0.24 0.27 0.32 0.35 0.29 0.22 0.4 0.48 0.52 1 0.28 0.41
PRDX6 0.32 0.36 0.18 0.37 0.27 0.24 0.41 0.33 0.19 0.27 0.33 0.28 1 0.38
RNASEH1 0.18 0.15 0.1 0.23 0.17 0.22 0.34 0.15 0.35 0.28 0.44 0.41 0.38 1
1
0.78
0.56
0.33
0.11
-0.11
-0.33
-0.56
-0.78
-1
D
Dysfunction
3
2
1
0
-1
-2
Low
High

Sfig. 6

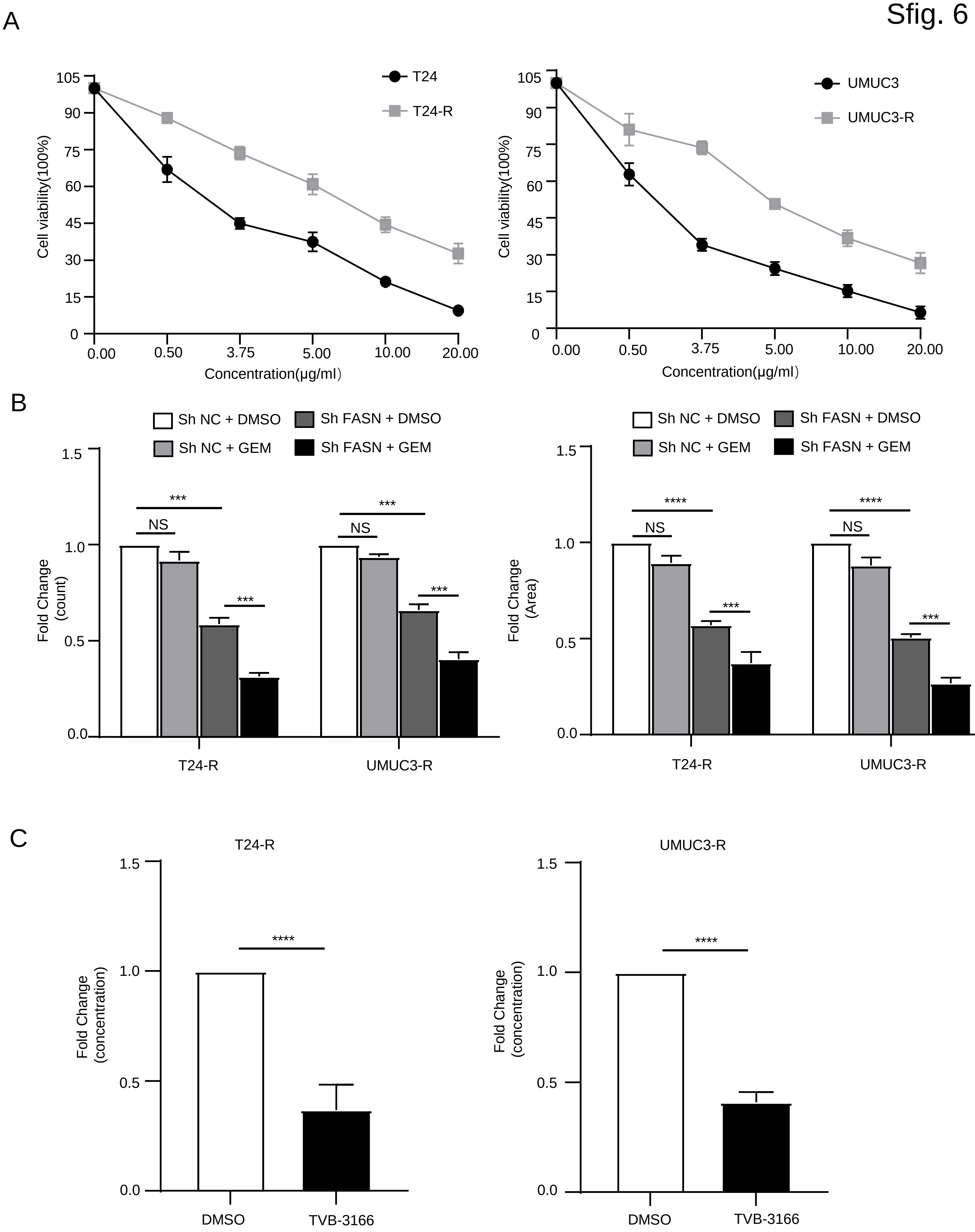
A
T24
T24-R
Cell viability(100%)
Concentration(μg/ml)
UMUC3
UMUC3-R
B
Sh NC + DMSO
Sh FASN + DMSO
Sh NC + GEM
Sh FASN + GEM
Fold Change (count)
Fold Change (Area)
NS
***
****
T24-R
UMUC3-R
C
T24-R
UMUC3-R
Fold Change (concentration)
****
DMSO
TVB-3166

Supplementary Figure Legends

SFigure1. (A) Gene Ontology (GO) enrichment analysis for differentially expressed genes (DEGs). Adjusted $p < 0.01$ and $p < 0.05$ were considered significant. **(B， C, and D)** Consensus clustering distribution function (CDF), area under CDF curve increment, and tracking plot for k = 2 to 9. **(E)** Individuals of principal component analysis (PCA) with two groups.

SFigure2. (A) The correlation of the OS-related key RM-DEGs. **(B)** MRS was positively correlated with Clinical stage and TNM stage, but not with age and gender. Student's t test; *, $p < 0.05$; **, $p < 0.01$; ***, $p < 0.001$.

SFigure3. (A) The distribution of risk scores in GSE69795 and GSE31684 database based on median of RM-RS. Blue represents low RM-RS subgroup, while red represents high RM-RS subgroup. **(B)** The Overall Survival (OS) distribution of patients(blue) or recurred (red) in subgroups. **(C)** The heatmap of RM-RM 7 component genes expression in GSE69795 and GSE31684 database, including RM-RS and clinical features. **(D)** Goat-IgG, Rabbit-IgG and Mouse-IgG as negative controls for immunohistochemistry with specific isoform-specific antibodies.

SFigure4. (A) Gene Ontology (GO) analysis for exploring molecular function and biological process involved in differentially expressed genes (DEGs) of two risk subgroups. **(B)** Metabolic pathways with significant differences between the two risk subgroups. *, $p < 0.05$; **, $p < 0.01$; ***, $p < 0.001$.

SFigure5. (A) Tumor microenvironment (TME) related gene sets enriched in high and low risk subgroups ($p < 0.05$). **(B)** Tumor microenvironment (TME) cells with significant differences in different RM-RS subgroups based on EPIC and MCPCOUNTER algorithm. **(C)** Correlation heatmap of RM-RS and cancer-associated fibroblasts (CAFs) marker gene expression. **(D)** Comparison of Dysfunction score in

different risk subgroups.

SFigure6. (A) Establishment of gemcitabine-resistant cell lines. **(B)** The tumorigenic ability of single cells under all conditions were determined by Colony formation assay. The histogram showed the comparison of the count and area of tumors. **(D)** Enzyme-linked immunosorbent assay (ELISA) was used to determine the FASN content of T24-R cells and UMUC3-R cells treated with TVB-3166(1μmol).
